# The Multifaceted Role of Regulatory T Cells in Breast Cancer

**DOI:** 10.1146/annurev-cancerbio-042920-104912

**Published:** 2020-12-04

**Authors:** Kevin Kos, Karin E. de Visser

**Affiliations:** 1 Division of Tumor Biology and Immunology, Oncode Institute, Netherlands Cancer Institute, 1066 CX Amsterdam, The Netherlands; 2 Department of Immunology, Leiden University Medical Center, 2333 ZA Leiden, The Netherlands

**Keywords:** regulatory T cell, breast cancer, metastasis, tumor microenvironment, immunosuppression

## Abstract

The microenvironment of breast cancer hosts a dynamic cross talk between diverse players of the immune system. While cytotoxic immune cells are equipped to control tumor growth and metastasis, tumor-corrupted immunosuppressive immune cells strive to impair effective immunity and promote tumor progression. Of these, regulatory T cells (T_regs_), the gatekeepers of immune homeostasis, emerge as multifaceted players involved in breast cancer. Intriguingly, clinical observations suggest that blood and intratumoral T_regs_ can have strong prognostic value, dictated by breast cancer subtype. Accordingly, emerging preclinical evidence shows that T_regs_ occupy a central role in breast cancer initiation and progression and provide critical support to metastasis formation. Here, T_regs_ are not only important for immune escape but also promote tumor progression independent of their immune regulatory capacity. Combining insights into T_reg_ biology with advances made across the rapidly growing field of immuno-oncology is expected to set the stage for the design of more effective immunotherapy strategies.

## 1 The Immune System: A Double-Edged Sword in Cancer

Tumors are complex entities consisting of not just cancer cells but also a variety of nonmalignant cell types. The local niche within and surrounding tumors is collectively described as the tumor microenvironment (TME), which can profoundly impact the development and progression of cancer (Blomberg et al. 2018, Garner & de Visser 2020, Hanahan & Weinberg 2011). It is now clear that the TME is not a static element of tumors, but its composition and functional state are highly diverse between cancer types, subtypes, and even individual tumors. In the past several decades, the immunological component of the TME has been studied extensively, with a focus on answering the central question: How can tumors develop in the context of a functional immune system? Addressing this fundamental question is essential to fully exploit the immune system for the treatment of cancer.

Breast cancer is perhaps one of the most studied cancer types in the context of the TME. Although survival rates for breast cancer patients are steadily increasing, it is still the leading cause of cancer-related deaths in women worldwide (Bray et al. 2018, DeSantis et al. 2019). The vast majority of breast cancer–related mortality is due to the incurable metastatic stage of the disease. Clearly, understanding, preventing, and treating metastatic breast cancer are unmet needs. As such, mechanistic insights into the complex interactions of key players in the TME could pave the way for novel innovative treatments and improved patient stratification.

Clinical studies have exposed a dual role of the immune system in breast cancer. For example, tumor-associated macrophages (TAMs) are associated with invasion, metastasis, and a worse prognosis (Qiu et al. 2018), while tumor-infiltrating lymphocytes (TILs) are associated with a favorable prognosis (Denkert et al. 2018). To understand this duality, one must realize that cancers host a plethora of immune cell subsets, such as lymphocytes, various myeloid cells, and innate lymphoid cells, to which both pro- and antitumorigenic functions have been attributed (Blomberg et al. 2018). Although immune cells such as CD8+ T cells and natural killer (NK) cells have the molecular gear to recognize and eradicate malignant cells, they often encounter a highly immuno-suppressive environment in tumors, which blunts effective antitumor immunity. This milieu is characterized by widespread expression of immune checkpoint receptors, inhibitory cytokines, hypoxia, and low levels of nutrients, all of which restrain the recruitment and function of cytotoxic immune cells (Binnewies et al. 2018). Importantly, lymphocytes and tumor-associated myeloid cells including macrophages, neutrophils, and monocytes profoundly contribute to the creation of this immunosuppressive environment, as well as to the systemic immunosuppression that often accompanies primary tumor growth and further potentiates cancer progression by facilitating immune escape (Garner & de Visser 2020).

A key orchestrator of immunosuppression is the CD4^+^ regulatory T cell (T_reg_), which since its discovery has been in the crosshairs of cancer immunology research (Plitas & Rudensky 2020, Yano et al. 2019). T_regs_ can be abundantly present in primary breast tumors and metastases (Syed Khaja et al. 2017). Still, their exact impact and relevance to breast cancer progression have proven challenging to uncover due to the complexities of immune cell cross-talk and metastatic disease. Recently, fundamental and preclinical research has provided exciting new insights into the biology of T_regs_ in breast cancer. This comes at an important time, as initial results for immune checkpoint inhibitors in breast cancer have been relatively disappointing (Planes-Laine et al. 2019). The expanding use of these drugs for the treatment of breast cancer therefore necessitates a comprehensive understanding of immunosuppressive T_regs_: Are we pulling the right strings? In this review, we therefore explore and discuss the current knowledge, challenges, and clinical use of T_regs_ in breast cancer.

## 2 T_Regs_: Gatekeepers of Immune Homeostasis

### 2.1 The Discovery and Biology of T_regs_

The immune system is a sophisticated defense network, evolved to withstand innumerable pathogenic challenges at any anatomical location. To do so, complex cellular interactions coordinate pathogen recognition, immune cell activation, and the execution of effector programs. In order to return to or maintain homeostasis, immunosuppressive signals are essential to dampen immune responses to prevent pathological immune responses such as chronic inflammation or autoimmunity. A key cell type involved in this process is the T_reg_. The importance of T_regs_ in immune tolerance has become evident through characterization of so-called scurfy mice, which suffer from a severe lethal autoimmune syndrome characterized by inflamed skin, red eyes, enlarged lymphoid organs, and early death (Russell et al. 1959). Scurfy mice were first reported in 1949, but it was not until the early 2000s that a mutation in the *Foxp3* gene, and consequential loss of T_regs_, was identified as a direct cause for the severe immune pathology (Brunkow et al. 2001). Further research showed that FOXP3 is the master transcription factor (TF) for the previously identified specialized immunosuppressive CD4^+^ CD25^+^ T lymphocytes, now known as T_regs_ (Fontenot et al. 2003, Hori et al. 2003). Since then, it has become clear that reduced T_reg_ numbers or impaired T_reg_ functionality stands at the basis of autoimmune and inflammatory diseases such as diabetes, multiple sclerosis, and inflammatory bowel disease (Bluestone et al. 2015, Leonard et al. 2017). In contrast, their activation and accumulation in tumors are considered detrimental, as we explore below in depth.

T_regs_ utilize several strategies to antagonize both adaptive and innate immunity. Among these, the release of immunosuppressive mediators, such as IL-10, TGF-β, and adenosine, and high expression of immunomodulatory receptors, such as CTLA-4, PD-L1, and LAG-3, are well established aspects of T_reg_ functionality that can interfere with the propagation of immune responses (Josefowicz et al. 2012a, Lucca & Dominguez-Villar 2020, Yano et al. 2019). Scavenging of IL-2 from the environment and killing of effector T cells by the release of granzymes additionally contribute to immunosuppression (Loebbermann et al. 2012, Vignali et al. 2008). Combined, these mechanisms can be employed to restrain dendritic cell (DC) function or directly inhibit cytotoxic cells (Vignali et al. 2008). The exact effector program that is engaged is highly dependent on the tissue and nature of the immune response (Josefowicz et al. 2012a, Koizumi & Ishikawa 2019). Emerging evidence shows that T_regs_ can acquire expression of T helper (T_h_) subset TFs, such as T-bet, GATA3, and RORγT, which direct their function towards suppression of Th cells of that particular subset (Josefowicz et al. 2012a, Koizumi & Ishikawa 2019). For example, T_regs_ expressing the T helper type 1 (T_h_1) TF T-bet are important for suppressing T_h_1-mediated inflammation but cannot suppress T_h_2 or T_h_17 responses (Chinen et al. 2016).

### 2.2 Two Flavors of FOXP3^+^ T_regs_

In vivo, two distinct populations of FOXP3^+^ T_regs_ are defined, based on their ontogeny and stability: thymically developed (natural) T_regs_ and extrathymically developed (peripheral or induced) T_regs_. Thymic T_regs_ (tT_regs_) represent a dedicated lineage with stable expression of FOXP3 and affinity for self-antigen. The generation of tT_regs_ occurs through a unique developmental program in the thymus, based on a delicate balance of T cell receptor (TCR) affinity and antigen specificity of CD4^+^ progenitor cells (Legoux et al. 2015, Malhotra et al. 2016, Moran et al. 2011). Through this program, tT_regs_ are equipped with TCRs biased towards recognition of tissue-restricted self-antigens, which enable the suppression of immune responses directed towards host peptides upon activation via their TCRs (Jordan et al. 2001, Kieback et al. 2016, Sakaguchi et al. 2008).

Unlike tT_regs_, peripheral T_regs_ (pT_regs_) are extrathymically generated in the periphery from nonregulatory FOXP3^−^ CD4^+^ T cells. A crucial element of pT_reg_ differentiation is their dependence on TGF-β signaling, which in FOXP3^−^ CD4^+^ T cells induces the interaction of SMAD2/3 with an intronic enhancer in the *FOXP3* locus, CNS1 (Kanamori et al. 2016, Marie et al. 2005, Zheng et al. 2010). pT_regs_ have unstable FOXP3 expression and lack the characteristic demethylation of the intronic element CNS2 observed in tT_regs_, which is essential for T_reg_ stability during proliferation (Kanamori et al. 2016, Lee & Lee 2018). In addition, pT_regs_ display a TCR repertoire that recognizes foreign antigens, parallel to conventional CD4+ T cells (Curotto de Lafaille & Lafaille 2009). As such, pT_regs_ have been found to play important roles at barrier sites, including the gut, lungs, and placenta, to mitigate inflammatory responses in response to foreign, but harmless, environmental, dietary, and microbial antigens (Esterházy et al. 2019, Josefowicz et al. 2012b, Kalekar et al. 2016, Soroosh et al. 2013).

The specific contributions of either tT_regs_ or pT_regs_ in cancer remain elusive, as to date no genuine phenotypic or functional marker has been discovered to distinguish both T_reg_ subtypes in vivo (Szurek et al. 2015). Instead, the ontogeny of T_regs_ in human cancer samples can be assessed ex vivo either via TCR repertoire sequencing or via epigenetic analysis of the CNS2 element in the *FOXP3* gene, which is demethylated in tT_regs_ but mostly methylated in pT_regs_. As most studies on T_regs_ do not distinguish between tT_regs_ and pT_regs_, below we refer to these cells as T_regs_, unless stated otherwise.

Now, nearly two decades after their discovery, the extent of T_reg_ functionality appears astonishingly diverse. T_regs_ play critical roles in tissue regeneration and repair, intestinal regulation of the microbiome, hair morphogenesis, metabolic homeostasis, pregnancy, and cancer (Josefowicz et al. 2012a, Sharma & Rudra 2018). However, it is less clear which mechanisms are engaged in the context of breast cancer progression and metastasis. Therefore, below we first review the evidence for the clinical relevance of T_regs_ in breast cancer.

## 3 Clinical Significance of T_Regs_ in Breast Cancer

### 3.1 Prognostic Value of T_regs_ in Breast Cancer

The discovery in 2001 that CD4^+^ CD25^+^ immunosuppressive cells can be found in the blood of healthy individuals (Baecher-Allan et al. 2001) kick-started research into the presence and behavior of these cells in cancer patients. In the following years, it was reported that CD4^+^ CD25^+^ T cells are increased in blood and tumors of patients with a variety of cancers, including breast, pancreatic, ovarian, and non-small-cell lung cancer (NSCLC) (Mougiakakos et al. 2010). However, as CD25 expression is not restricted to T_regs_, but can also be expressed by effector T cells, it was not until the discovery of FOXP3 as a unique marker of T_regs_ (Fontenot et al. 2003, Hori et al. 2003) and the development of reliable monoclonal antibodies that the presence of T_regs_ could be convincingly demonstrated in human cancers (Bates et al. 2006, Roncador et al. 2005). Since then, many studies have investigated the association between the presence of intratumoral T_regs_ and patient survival and therapy response in breast cancer (Table 1).

Despite an extensive body of literature, the clinical significance of T_regs_ in breast cancer remains controversial due to conflicting results among studies (Table 1). A key challenge in interpreting these studies is that the prognostic value of T_regs_ seems to differ by molecular breast cancer subtype. These subtypes are broadly defined on the basis of tumoral expression of the estrogen and progesterone hormone receptors (HR^+^) and the growth factor receptor HER2 or the absence of these [triple-negative breast cancer (TNBC)] (DeSantis et al. 2019). Several meta-analyses published over the last few years have showed that high FOXP3 TILs in HR^+^ breast tumors correlate with poor survival, high grade, and lymph node involvement (Jiang et al. 2015, Wang et al. 2016, Zhou et al. 2017). However, multivariate Cox regression on patient outcomes, including adjustments for tumor size, grade, and lymph node stage, has revealed that FOXP3 TILs are not an independent prognostic factor in HR^+^ breast tumors (Liu et al. 2014, Mahmoud et al. 2011). Whether T_regs_ are causally involved in the differentiation of high-grade tumors, lymph node metastasis, and poor prognosis cannot be determined from these descriptive analyses. In contrast to HR^+^ breast cancer, FOXP3 TILs strongly correlate with a favorable prognosis in HR^−^ and TNBC subtypes (Jiang et al. 2015, Mahmoud et al. 2011, Tsang et al. 2014, West et al. 2013). Here, T_reg_ infiltration is strongly associated with high CD8^+^ and Th cell infiltration, perhaps reflecting a T cell–permissive environment (Seo et al. 2013). This is further supported by the observation that T_regs_ are not associated with prognosis in triple-negative tumors with low CD8^+^ T cell infiltration (West et al. 2013). In conclusion, T_regs_ correlate with disease outcomes in a subtype-dependent manner, but future preclinical research is needed to uncover the mechanistic link between T_regs_ and breast cancer subtypes.

### 3.2 Predictive Value of T_regs_ in Cancer Immunotherapy

Novel therapeutics targeting immune checkpoints such as PD-1/PD-L1 and CTLA-4 are transforming the treatment landscape across cancer types (Wei et al. 2018). In order to maximize efficacy, numerous studies are currently evaluating predictive biomarkers and novel treatment combinations (Kim et al. 2019). Importantly, T_regs_ can be direct targets of these treatments due to their high expression of immune checkpoint molecules (Togashi et al. 2019). While the use of immunotherapy in breast cancer is still in its infancy, research in other cancer types has revealed the potential predictive significance of T_regs_ in the context of PD-1/PD-L1 blockade. For example, PD-1 blockade has been associated with disease progression in gastric cancer (GC) patients via the activation and expansion of intratumoral PD-1^+^ T_regs_ (Kamada et al. 2019). Accordingly, PD-1 expression by intratumoral T_regs_ was found to predict resistance to anti-PD-1 therapy in multiple NSCLC and GC patient cohorts (Kumagai et al. 2020). In addition, high intratumoral T_reg_ proliferation in response to anti–PD-1 therapy has been linked to recurrence (Huang et al. 2019). Finally, PD-L1-mediated expansion of pT_regs_ is an important immunosuppressive axis in glioblastoma (DiDomenico et al. 2018). In recent years, the first trials investigating the efficacy of immune checkpoint blockade (ICB) in metastatic TNBC have been published, with a strong focus on PD-1/PD-L1 blockade (Adams et al. 2019, Dirix et al. 2018, Emens et al. 2019, Nanda et al. 2016, Planes-Laine et al. 2019, Schmid et al. 2018, Voorwerk et al. 2019). Although clinical benefit is observed for a small proportion (approximately 5–20%) of breast cancer patients, emerging evidence shows that selecting patients based on immune parameters such as a high TIL score and high PD-L1 expression may modestly improve response rates (Emens et al. 2019). Up until now, T_regs_ have not been specifically reported to be correlated with efficacy in these early studies. As such, research in the coming years should clarify whether T_regs_ are predictive for the success of PD-1/PD-L1-based treatments in breast cancer.

### 3.3 Qualitative Clinical Assessment of T_regs_ in Breast Cancer

Besides quantification of intratumoral T_regs_, there is a growing body of evidence indicating that a more in-depth qualitative assessment of T_regs_, including information on phenotype, functional state, and immune cell cross talk, may be important for disease outcome. For example, recent reports have shown that intratumoral T_regs_ from breast cancer patients display an activated phenotype with high expression of CD25, CTLA-4, and PD-1 and exert immunosuppressive activity (Gobert et al. 2009, Plitas et al. 2016, Syed Khaja et al. 2017). In one of these studies, the transcriptome of T_regs_ from 105 treatment-naïve breast cancer patients was analyzed (Plitas et al. 2016). The chemokine receptor CCR8 was identified to be uniquely expressed by intratumoral T_regs_, but not by T_regs_ isolated from breast tissue and blood from healthy donors. CCR8^+^ T_regs_ were found to be highly proliferative and enriched in high-grade tumors. Strikingly, while intratumoral T_reg_ abundance based on *FOXP3* mRNA expression did not correlate with clinical features, stratifying patients based on the *CCR8*:*FOXP3* ratio in the tumor revealed a strong correlation with poor survival in patients (Plitas et al. 2016). These findings illustrate that in-depth analysis of intratumoral T_regs_ provides important information. As the patients in this cohort predominantly had HR^+^ tumors (74.3%), an important next step would be to validate these findings in HER2^+^ and TNBC subtypes, in which T_regs_ are associated with good prognosis (West et al. 2013).

Many studies have reported increased frequencies of T_regs_ in the peripheral blood of breast cancer patients across subtypes (Decker et al. 2012, Horlock et al. 2009, Liyanage et al. 2002, Perez et al. 2007, Wolf et al. 2003), indicating that breast tumors can systemically engage T_regs_. Still, their significance remained elusive for a long time until a recent in-depth analysis performed on T_regs_ isolated from the blood and tumors of breast cancer patients (Wang et al. 2019). It was found that a subpopulation of T_regs_ (FOXP3^hi^ CD45RA^neg^) (Miyara et al. 2009), comprising approximately 19% of the total T_reg_ population in the peripheral blood of patients, strongly resembles intratumoral T_regs_, based on phenotype, TCR repertoire, and CCR8 expression. This may suggest that intratumoral T_regs_ derive from FOXP3^hi^ CD45RA^neg^ T_regs_ in peripheral blood, or vice versa. These T_regs_ from blood had superior suppressive potential in vitro, compared to FOXP3^low^ CD45RA^pos/neg^ T_regs_. FOXP3^hi^ CD45RA^neg^ T_regs_ were found to be heterogeneous between patients in their signaling response to both immunosuppressive and inflammatory cytokines. High-T_reg_ responsiveness to immunosuppressive cytokines correlated with poor survival, whereas high responsiveness to inflammatory cytokines had the opposite effect (Wang et al. 2019). This exposes the potential clinical significance of T_regs_ in the peripheral blood of breast cancer patients, but also highlights how T_reg_ heterogeneity may potentially influence disease outcomes.

Over recent years, studies focusing on FOXP3 TILs have been moving from basic quantification analyses towards sophisticated in-depth characterization, yielding exciting new insights with prognostic and potential therapeutic implications. As we are starting to discover the characteristics of T_regs_ with tumor-promoting capabilities, mechanistic studies should investigate their functional roles in breast cancer progression, and whether their emergence can be therapeutically halted.

## 4 Mechanistic Understanding of T_Regs_ in Breast Cancer

### 4.1 The Context-Dependent Functional Role of T_regs_ in Breast Cancer Progression

Preclinical animal models are key to mechanistically understanding how T_regs_ impact breast cancer progression. An important tool to dissect T_reg_ function in these models is their systemic depletion, which can be achieved via two strategies. Firstly, antibody-based approaches deplete T_regs_ through targeting of cell-surface receptors that are highly expressed on T_regs_, including CD25, GITR, and FR4 (Arce Vargas et al. 2017, Coe et al. 2010, Yamaguchi et al. 2007). Secondly, the development of transgenic mice that express the diphteria toxin receptor (DTR) under control of *Foxp3* either via direct knockin (*Foxp3* ^DTR^ mice) or by its introduction using a bacterial artificial chromosome [DEREG (depletion of T_regs_) mice] has allowed for short-term inducible depletion of T_regs_ upon injection of diphteria toxin (DT) (Kim et al. 2007, Lahl et al. 2007). A transgenic mouse model for mammary tumorigenesis that is regularly used to study the biology of T_regs_ in breast cancer is the MMTV-PyMT (mouse mammary tumor virus–polyoma middle tumor-antigen) mouse model. T_regs_ have been shown to highly infiltrate mammary tumors of MMTV-PyMT mice, depending in part on CCR2 expression on T_regs_ (Loyher et al. 2016). Ablation of T_regs_ in *Foxp3* ^DTR^ mice with orthotopically transplanted MMTV-PyMT tumors drastically reduced tumor growth and pulmonary metastases (Bos et al. 2013). Mechanistically, IFNγ and CD4^+^ conventional T cells were required for the observed antitumor effect, which was independent of CD8^+^ T cells and NK cells. As proinflammatory signaling by myeloid cells was increased upon T_reg_ depletion, the authors of this study speculated that IFNγ-activated macrophages may contribute to antitumoral inflammation (Bos et al. 2013).

The observation that T_regs_ constrain antitumor immunity in tumors has been reported by others. For example, anti-CD25 treatment in mice inoculated with 4T1 cancer cells strongly reduced tumor growth, which correlated with an increase in DCs and effector CD8^+^ T cells in tumor-draining lymph nodes (TDLNs), suggesting that T_regs_ modulate DC function (Goudin et al. 2016). Indeed, it has been reported that T_regs_ can inhibit the expression of costimulatory ligands on DCs, thereby restraining CD8^+^ T cell activation and tumor clearance in a *Kras*-mutant model for pancreatic cancer (Jang et al. 2017). It would be of interest to investigate whether similar mechanisms are at play in breast cancer. Elimination of T_regs_ is not always sufficient to drive strong antitumor responses. For example, immunosuppressive T_regs_ were found to be highly enriched in inoculated TNBC T-11 tumors, but DT-based T_reg_ ablation only slightly slowed tumor growth. T_reg_ ablation did potentiate PD-1/CTLA-4-based immunotherapy, which correlated with an increase in IFNγ^+^ CD8^+^ T cells (Taylor et al. 2017). These findings suggest that T_regs_ can form an important barrier for immunotherapy-induced antitumor immunity, which has been reported before in preclinical inoculated melanoma and colon carcinoma tumors (Arce Vargas et al. 2017).

The studies above suggest that targeting T_regs_ in (breast) cancer models induces antitumoral inflammation that, sometimes in combination with immunotherapy, may unleash antitumor immune responses. However, therapeutic elimination of T_regs_ may trigger autoimmunity in cancer patients, particularly in combination with ICB. Thus, an important next step would be to define the context-dependent molecular mechanisms engaged by T_regs_ to enable precise targeting of relevant effector programs instead. A key challenge here is the apparent variability of the clinical significance of T_regs_ with breast cancer subtype, which necessitates studying these cells in clinically relevant mouse tumor models. Currently, the vast majority of murine breast cancer cell lines used for inoculation into mice and genetically engineered mouse models (GEMMs) for breast cancer give rise to estrogen receptor–negative (ER^−^) mammary tumors (Özdemir et al. 2018), whereas ~75% of human invasive breast cancers are ER^+^ (Bentzon et al. 2007). As T_regs_ have been particularly associated with a detrimental role in HR^+^ breast cancers, future research should ideally focus on the development and use of HR^+^ breast tumor models to uncover the subtype-dependent role of T_regs_ in breast cancer.

**Figure 1:**
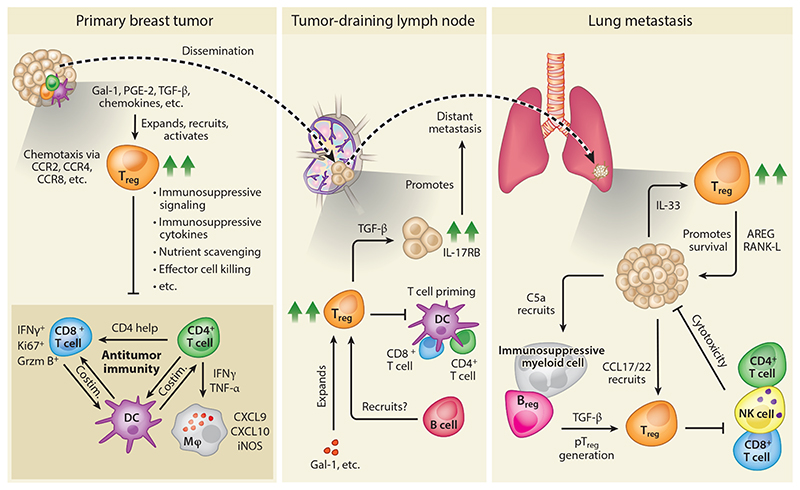
T_regs_ modulate their local environment to promote breast cancer progression. Tumor-derived factors such as chemokines, cytokines, and other mediators promote the accumulation and expansion of T_regs_ in primary breast tumors and metastatic niches. In the TME, T_regs_ constrain both innate and adaptive immune responses to counteract antitumor immunity. Mechanistically, T_regs_ can (among other effects) suppress the expression of costimulatory ligands on DCs, release inhibitory modulators that interfere with T cell activation, and are also equipped to induce apoptosis in effector cells (*left*). However, the effector mechanisms that are engaged in the context of the breast TME remain largely unknown. In addition, T_regs_ can enhance metastatic progression of cancer cells in tumor-draining lymph nodes (*middle*) and lungs (*right*) through tissue-specific mechanisms. These mechanisms include promoting tumor cell survival and migration via secretion of TGF-β, AREG, and RANK-L, as well as inhibiting cytotoxic effector cells. Abbreviations: B_reg_, regulatory B cell; costim., costimulation; DC, dendritic cell; Gal-1, galectin 1; Grzm B, granzyme B; iNOS, inducible nitric oxide synthase; M_φ_, macrophage; NK, natural killer; pT_reg_, peripheral T_reg_; TME, tumor microenvironment; T_reg_, regulatory T cell.

While T_regs_ can interfere with antitumor immunity in the context of established tumors (Figure 1), recent findings in spontaneously developing tumor models suggest that at the onset of neoplastic progression, T_regs_ may unexpectedly constrain protumoral inflammation. One study reported that DT-based ablation of T_regs_ during the early, noninvasive neoplastic phase in the MMTV-PyMT model accelerated the progression of noninvasive lesions into invasive tumors (Martinez et al. 2019). The elimination of T_regs_ resulted in the accumulation of macrophages in mammary glands and an induction of the T_h_2 cytokines IL-4 and IL-5, which have been reported to induce tumorigenic functions in macrophages (DeNardo et al. 2009). The CD44^+^ CD24^−^ mammary stem cell compartment was also found to be expanded, with increased colony forming capacity in vitro. Whether T_regs_ control mammary stem cell proliferation directly, or indirectly via the TME, remains to be addressed. In line with these findings, T_regs_ have also been reported to inhibit pancreatic carcinogenesis of neoplastic lesions in a *Kras*-mutant GEMM by repressing the recruitment of immunosuppressive myeloid cells (Zhang et al. 2020). These findings reinforce that T_regs_ are potent suppressors of inflammation in early stages of tumorigenesis, which has context-dependent effects on tumor progression. As T_regs_ have been found to expand in ductal carcinoma in situ (Bates et al. 2006), more research is needed to uncover whether these cells play a protective or detrimental role in precancerous breast cancer lesions.

Research on T_regs_ in other cancer types has revealed the versatile nature of these cells and has uncovered novel mechanisms of immune cell cross talk (Jang et al. 2017). For example, T_reg_-derived IL-10 and IL-35 can promote CD8^+^ T cell exhaustion in melanoma (Sawant et al. 2019). It is also becoming increasingly clear that T_regs_ can interact with a variety of myeloid cells to hamper antitumor immunity, including eosinophils, mast cells, macrophages, neutrophils, and basophils (Blatner et al. 2010, Zhou et al. 2018). T_regs_ were found to control intratumoral eosinophil and basophil infiltration, both of which can promote recruitment of CD8^+^ T cells, leading to tumor rejection of melanoma cell lines (Carretero et al. 2015, Sektioglu et al. 2017). In addition, T_regs_ indirectly maintain an immunosuppressive phenotype in TAMs by inhibiting the release of IFNγ in the TMEs of inoculated B16 and MC38 tumors (Liu et al. 2019). Up until now, these interactions have not been investigated in the context of breast cancer, illustrating that we have perhaps only scratched the surface on the effector functions of T_regs_ in breast cancer. Promisingly, a transcriptional signature specific for tumor-infiltrating T_regs_ has revealed remarkable similarity across tumor types in both human and mouse (Magnuson et al. 2018), suggesting that effector mechanisms may be shared across tumor types. Accordingly, the chemokine receptor CCR8 was identified as part of this signature, supporting previously discussed findings in human breast cancer (Plitas et al. 2016).

### 4.2 Mechanisms of Intratumoral Accumulation of T_regs_ in Breast Tumors

Three main hypotheses have been postulated to explain the accumulation of T_regs_ in breast tumors. Firstly, T_regs_ that circulate in peripheral blood and lymph nodes may migrate into the TME following chemokine gradients upon activation. Secondly, it has been hypothesized that tissue-resident T_regs_ locally expand in the TME. Finally, intratumoral conversion of conventional CD4^+^ T cells into pT_regs_ may represent an important mechanism for T_reg_ accumulation. Although these hypotheses are not mutually exclusive and may all contribute to T_reg_ accumulation, the migration hypothesis in particular has been supported by experimental evidence. Studies in humans and mice have shown that T_regs_ express a wide range of chemokine receptors that may facilitate intratumoral homing, of which CCR2, CCR4, CCR5, CCR8, CXCR3, and CXCR6 have been associated with breast cancer (Plitas et al. 2016, Yano et al. 2019). For example, CCR2^+^ T_regs_ accumulate in multiple tumor models, including the PyMT-MMTV model (Loyher et al. 2016). These cells display an activated phenotype and were found to be tumor-antigen specific in an OVA (ovalbumin)-expressing sarcoma cell line inoculation model. Specific ablation of CCR2 on T_regs_ strongly reduced intratumoral T_reg_ accumulation (Loyher et al. 2016). CCR2 was also found to be expressed by intratumoral T_regs_ in human breast tumors (Plitas et al. 2016). Others have reported high expression of CCR4 by T_regs_ in the blood of breast cancer patients, with migratory capabilities to CCL22 and CCL17 (Gobert et al. 2009). As discussed above, CCR8 has emerged as a chemokine receptor expressed uniquely by tumor-associated T_regs_ (Plitas et al. 2016, Wang et al. 2019) and has therefore gained attention as a potential therapeutic target. Anti-CCR8 monoclonal antibody (mAb) treatment of mice inoculated with CT26 colon carcinoma cells significantly reduced T_regs_ in tumors and enhanced intratumoral IFNγ expression (Villarreal et al. 2018). In contrast, others have shown that CCR8 may be redundant for intratumoral T_reg_ homing, as adoptively transferred Ccr8-knockout T_regs_ in mice inoculated with MC38 colon carcinoma cells did not display reduced potential of migrating into tumors (Magnuson et al. 2018). It has also been reported that autocrine production of CCL1, the ligand for CCR8, potentiates both T_reg_ proliferation and suppressive potential (Barsheshet et al. 2017), suggesting that CCR8 may play an important role in maintaining T_reg_-mediated immunosuppression, in addition to its chemotactic properties.

Accumulating evidence shows that intratumoral T_regs_ in breast cancer are transcriptionally distinct from T_regs_ in peripheral blood and lymph nodes and share gene expression profiles with mammary tissue–resident T_regs_ (Azizi et al. 2018, Plitas et al. 2016, Szabo et al. 2019). This suggests either that tissue-resident cells expand in tumors or that the local TME drives transcriptional adaption of cells migrating into the TME. It has been reported that intratumoral and healthy breast T_regs_ within patients showed relatively little overlap of their TCR repertoire, suggesting that intratumoral T_regs_ do not derive from resident cells (Plitas et al. 2016). In addition, Ki67 expression in T_regs_ of healthy breast tissue was found to be drastically lower than that in T_regs_ from tumor or blood. In line with the second notion, single-cell RNA sequencing (scRNA-seq) of T_regs_ of naïve mice revealed that T_reg_ migration from lymphoid to nonlymphoid tissues indeed induces a transcriptional program specifically tailored to the destined tissue (Miragaia et al. 2019). Furthermore, scRNA-seq of CD45^+^ cells sorted from human breast tumors, blood, and lymph nodes uncovered that intratumoral immune cells can acquire diverse phenotypes that are not found in circulation or normal tissue (Azizi et al. 2018). Here, five different T_reg_ clusters unique to the TME were identified that expressed gene sets related to activation, anti-inflammation, exhaustion, hypoxia, and metabolism. Together, these studies suggest that transcriptional adaptation of migratory T_regs_ in the TME may explain the transcriptomic resemblance between intratumoral and mammary tissue–resident T_regs_, although further TCR profiling and genetic tracing studies are needed to definitively confirm this.

Research on the accumulation of tT_regs_ versus pT_regs_ in cancer has been rather limited due to the complexities of distinguishing both T_reg_ subsets in vivo. Yet, local induction of pT_regs_ in the TME may in fact be an important mechanism of immunosuppression, as TGF-β is abundantly expressed in cancers (Batlle & Massagué 2019). However, analysis of T_regs_ in human glioma, melanoma, and lung cancer samples did not reveal a substantial contribution of pT_regs_ to the total intratumoral T_reg_ pool (Ahmadzadeh et al. 2019, Akimova et al. 2017, Lowther et al. 2016, Plitas et al. 2016). For example, one study found that the overlap between TCR clonotypes of FOXP3^+^ and FOXP3^−^ CD4^+^ T cells obtained from six melanoma tumors was 0.5–13.2%, indicating that a relatively small proportion of T_regs_ may have been pT_regs_. However, others have attributed important roles to pT_regs_ in murine cancer models (Alonso et al. 2018, Olkhanud et al. 2011, Schreiber et al. 2012, Su et al. 2017). One of these reports provided indications of their presence in the TME of breast cancer patients (Su et al. 2017). TCR repertoire analysis on CD4^+^ T cells from tumor, blood, and lymph nodes of five breast cancer patients revealed that tumor-infiltrating T_regs_ are most similar to naïve CD4^+^ T cells from tumor and blood, suggesting intratumoral conversion. By using the MDA-MB-231 TNBC cell line in humanized mice, these researchers further showed that TAM-secreted CCL18 specifically recruits naïve CD4^+^ T cells, but not T_regs_, via PITPNM3, into the TME. Here, these naïve CD4^+^ T cells were capable of converting into FOXP3^+^ T_regs_, via unknown mechanisms. Blocking CCL18 in tumor-bearing mice reduced intratumoral T_reg_ numbers and inhibited tumor growth (Su et al. 2017). As data on the role of pT_regs_ in breast cancer are still limited, future studies should focus on expanding these findings in a larger cohort of patients.

It is now well established that T_regs_ have various ways to accumulate in primary tumors. However, breast cancer survival is largely dictated by the extent of metastatic disease. Thus far, we have mostly discussed research on T_regs_ in breast cancer in the context of primary tumors, raising questions on the link between primary tumors and metastasis. Can T_regs_ impact metastasis formation from within the primary tumor? Or do circulating or tissue-resident T_regs_ induce a systemic immunosuppressive axis that impacts metastasis formation?

### 4.3 Impact of T_regs_ on Metastatic Progression

Primary cancer cells must progress through a multistep process in order to successfully metastasize. This so-called metastatic cascade consists of tumor cell invasion, intravasation, survival in the circulation, extravasation, and outgrowth in a foreign, hostile environment, all while evading destruction by the immune system (Blomberg et al. 2018). Prior to metastatic spread, tumor-derived systemic factors can even further potentiate metastasis by instructing (immature) myeloid cells to establish a premetastatic niche (Kitamura et al. 2015). T_regs_ may be involved in all steps of the metastatic cascade through mechanisms both dependent and independent of their immune-regulatory function. However, progress toward understanding their impact on the metastatic cascade is hampered by the limited availability of preclinical models that realistically recapitulate metastasis (Gómez-Cuadrado et al. 2017). Cancer cell line–based mouse models fail to fully recapitulate the chronic and systemic inflammation that underlies de novo tumor development, progression, and metastasis (Kersten et al. 2017). In addition, research in both 4T1 and PyMT models has shown that T_reg_ depletion reduces primary tumor growth (Bos et al. 2013, Liu et al. 2016), which may obscure mechanisms at play during the metastatic cascade. Indeed, using the 4T1 model, it was recently shown that control of primary tumor growth following T_reg_ depletion subsequently led to control of metastatic disease through the induction of protective immunity (Hughes et al. 2020). These data suggest that the potential direct effects of T_regs_ on the metastatic cascade are masked in tumor models that are responsive to T_reg_ depletion in a primary setting. Nevertheless, several studies have revealed that tumor-induced (systemic) activation of T_regs_ can contribute to metastatic progression (Figure 1). This activation can be mediated via the release of various tumor-derived soluble factors, such as prostaglandins, complement factors, and β-galactoside-binding proteins (Dalotto-Moreno et al. 2013, Karavitis et al. 2012, Vadrevu et al. 2014). For example, tumor-secreted galectin-1 was reported to enhance systemic expansion of T_regs_ and their suppressive potential, resulting in increased lung metastases in mice bearing inoculated 4T1 mammary tumors (Dalotto-Moreno et al. 2013). Others showed that overexpression of COX2 in inoculated TM40D mammary tumors enhanced bone metastasis, which correlated with increased recruitment of T_regs_ into the primary tumor (Karavitis et al. 2012). In addition to factors released by the primary tumor, the local (pre)metastatic niche can also play an important role in the activation and recruitment of T_regs_. For example, IL-33 and CCL17 have both been reported to be released in metastatic foci in the lungs of 4T1 tumor–bearing mice, leading to the accumulation of T_regs_ that express the receptor for these molecules, thereby promoting metastasis (Halvorsen et al. 2019, Olkhanud et al. 2009).

Various tumor-driven pathways exist to systemically engage T_regs_ to the benefit of metastatic spread. An underlying question remains how T_regs_ mechanistically contribute to metastasis. Interestingly, T_regs_ have been found to directly contribute to metastasis of the 4T1 and MT2 cell lines in mice by promoting tumor cell survival via the release of Rankl and Areg (Halvorsen et al. 2019, Tan et al. 2011). In addition, in line with their immunomodulatory properties, the prometastatic function of T_regs_ has been linked to inhibition of cytotoxic immune cells. Indeed, T_reg_-mediated inhibition of NK cells has been associated with increased pulmonary metastasis in the 4T1 model (Biragyn et al. 2013). Others found that neoadjuvant ablation of T_regs_ in 4T1 tumor–bearing *Foxp3* ^DTR^ mice almost completely abolished the formation of lung metastases, which was dependent on both CD4^+^ and CD8^+^ T cells but not NK cells (Liu et al. 2016). Of note, only neoadjuvant, and not adjuvant, T_reg_ depletion increased the systemic frequency and activation of tumor-specific CD8^+^ T cells (Liu et al. 2016). It has not been addressed whether CD4^+^ T cells directly engage in tumor cell killing in the absence of T_regs_, or whether they perhaps provide essential help for CD8^+^ T cell activation. The superiority of neoadjuvant over adjuvant targeting of T_regs_ suggests a role for T_regs_ in early stages of metastasis, which is supported by observations in breast cancer patients that T_reg_ accumulation associates with metastasis formation in draining lymph nodes (Faghih et al. 2014, Jiang et al. 2015, Núñez et al. 2020).

Several clinical studies have reported that high T_reg_ infiltration in primary tumors and sentinel lymph nodes is associated with the occurrence of lymph node metastasis (Table 1), but mechanistic data are limited. So far, one study has linked intranodal T_regs_ to breast cancer progression in mice. Here, in a 4T1 model, T_reg_-derived TGF-β1 induced IL-17RB in cancer cells in TDLNs (Huang et al. 2017). IL-17RB was found to potentiate the metastatic and colony-forming potential of cancer cells via NF-κB, which enhanced distant metastasis. Interestingly, analysis of IL-17RB expression in lymph node metastasis and matched tumors of breast cancer patients confirmed that IL-17RB is increased in lymph nodes and correlates with FOXP3 frequency (Huang et al. 2017). This study revealed that the TDLNs in breast cancer can function as a gateway to distant metastasis, with T_regs_ corrupted by the primary tumor. These findings raise the question whether T_regs_ are also involved in cancer cell dissemination to the draining lymph node. It has recently been reported that B cells promote metastasis to draining lymph nodes in the 4T1 and MMTV-PyMT models via the release of HSP4A-binding antibodies that directly promote tumor cell migration (Gu et al. 2019). Interestingly, B cell depletion did significantly reduce tumor-induced T_reg_ accumulation in TDLNs. In line with these findings, it has previously been reported that regulatory B cells that accumulate in 4T1 tumor–bearing mice can induce pT_regs_ in a TGF-β-dependent manner (Olkhanud et al. 2011), revealing an interesting cross talk between T_regs_ and B cells in breast cancer metastasis.

## 5 Future Prospects

T_regs_ have taken an increasingly important position in our understanding of the immune system in breast cancer. Preclinical research has revealed ingenious mechanisms employed by breast tumors to seize control of T_regs_ for their own benefit. In parallel, in-depth characterization of T_regs_ beyond traditional FOXP3 scoring in human samples is paving the way to advance the prognostic and predictive value of T_regs_ in the clinic. Here, future efforts should focus on further defining the heterogeneity of T_regs_ and evaluate which features of T_regs_ are instrumental for disease progression, while also expanding current findings to HR^−^ subtypes of breast cancer where T_regs_ are associated with a good prognosis. As the use of immunomodulatory drugs is gaining momentum in the clinic, interrogating these observations in the context of immunotherapy is also an important next step.

The context dependency under which T_regs_ operate should also be increasingly taken into account in preclinical research. Until now the majority of research has been performed in a limited number of (cell line–based) breast cancer models, often with unclear translatability to human disease. An important challenge to address here is that breast cancer patients suffer from metastatic spread to a broad spectrum of anatomical locations, while experimental metastasis in animal models is often limited to the lungs. A crucial next step is therefore to validate preclinical findings in murine models that have increased translatability, in terms of both cancer subtype and metastasis formation. To achieve this, one must realize that the interaction between the immune system and cancer may even be more complex than initially assumed. We are only now beginning to understand that the genetic makeup of tumors may profoundly impact their accompanying TME (Wellenstein & de Visser 2018). In addition, in-depth analyses of 168 metastatic and primary tumor samples from 10 breast cancer patients has revealed that the composition of metastatic TMEs within patients is heterogeneous, even within particular organs. Moreover, the expression of genes encoding immunomodulatory proteins such as PD-1 and PD-L1 differs across metastases within individual patients (De Mattos-Arruda et al. 2019). These complexities of human metastatic disease illustrate the need for accurate models of metastasis.

Ultimately, these fundamental insights into the role of T_regs_ in breast cancer progression could form the basis for therapeutic intervention. As such, several early phase clinical trials have evaluated the FDA (US Food and Drug Administration)-approved mAb daclizumab (anti-CD25) in combination with cancer vaccines in metastatic melanoma and breast cancer (Jacobs et al. 2010, Rech et al. 2012). FOXP3^+^ CD4^+^ T cells in peripheral blood were found to be reduced upon daclizumab treatment, but no significant clinical benefit was observed. However, daclizumab does not induce antibody-dependent cytotoxicity (ADCC), which others have suggested to be essential for intratumoral T_reg_ depletion and therapeutic efficacy (Arce Vargas et al. 2017, Rech et al. 2012). Recently, an optimized ADCC-inducing anti-CD25 antibody showed superior intratumoral T_reg_ depletion and induced CD8^+^ T cell–mediated tumor rejection in combination with anti-PD-1 therapy in preclinical models (Arce Vargas et al. 2017). Alternatively, intratumoral injection of CD25-targeting immunotoxins also potently depletes intratumoral T_regs_, leading to CD8^+^ T cell–mediated tumor regression of inoculated 66c14 breast cancer tumors (Onda et al. 2019). Importantly, these preclinical results suggest that effector T cell responses are not necessarily negatively impacted by CD25-based depletion, which may set the stage for clinical trials evaluating this new generation of T_reg_-targeting strategies. In addition to T_reg_ depletion, blocking of their intratumoral recruitment, conversion, or important effector mechanisms may be alternative future approaches to interfere with T_reg_-mediated modulation of breast cancer (Plitas & Rudensky 2020).

In conclusion, recent research has revealed T_regs_ as important modulators of breast cancer progression and metastasis, and exciting advancements in clinical analysis have improved the prognostic and predictive significance of these cells and the therapeutic potential of targeting them. The use of GEMMs that closely mimic the diversity and the stepwise progression of human breast cancer subtypes will propel our understanding of T_reg_ biology to a higher level and deepen our knowledge of underlying mechanisms. This knowledge could help researchers take full advantage of novel immunomodulatory drugs that may take the stage in breast cancer treatment.

## Figures and Tables

**Table 1:** Prognostic significance of FOXP3 TILs across breast cancer subtypes

Subtype analyzed	Patients (*n*)	Correlations with high FOXP3 TILs:			Reference
		Prognosis	Subtype	Clinical features	
DCIS	62	Poor (univariate)	DCIS^d^	ND	Bates et al. 2006
ER^−^	77	No effect	ER^−^	High grade, LN met^+^	
ER^+^	148	Poor (univariate)			
ER^−^	364	No effect	ER^−^; HER2^+^; basal	High grade, LN met^+^, large tumor size	Mahmoud et al. 2011
ER^+^	982	Poor (univariate)^a^			
Mixed	398	Poor (multivariate)	ER^−^; HER2^+^; basal	High grade	Yan et al. 2011
Mixed	1,270	Poor (multivariate)	ER^−^; PR^−^; HER2^+^	High grade	Liu et al. 2011
Mixed	72	Poor (univariate)^a^	NS	LN met^+^, p53^+^, Ki67^+^	Kim et al. 2013
Mixed	90	Poor (multivariate)	ER^−^; HER2^+^	High grade	Takenaka et al. 2013
Mixed	90	Poor (univariate)^a^	HER2^+^	High grade, LN met^+^, large tumor size	Maeda et al. 2014
Mixed	498	Poor (univariate)^a^	HER2^+^; TNBC	High *γδ* T cell	Allaoui et al. 2017
Mixed	118	Poor (univariate)	ND	High grade, LN met^+^, Ki67^+^, tumor nest	Peng et al. 2019
TNBC	86	Favorable (multivariate)	ND	LN met^+^	Lee et al. 2013
ER^−^ HER2^−^	175	Favorable (univariate)	NS	High grade, high CD8^+^, young age	West et al. 2013
ER^−^ HER2^+^		No effect			
ER^+^	2,166	No effect (multivariate)^b^	ER^−^; HER2^+^; basal	High grade, LN met^+^, High CD8^+^, young age	Liu et al. 2014
ER^−^ HER2^+^	250	No effect (multivariate)^c^			
Basal	330	Favorable (multivariate)			
ER^+^	554	ND	ER^+^	ND	Tsang et al. 2014
ER^−^ HER2^+^					
Mixed	218	No effect	ND	High grade, high CD8^+^, high PD1^+^	Sun et al. 2014
TNBC	101	No effect	ND	High CD8^+^	Miyashita et al. 2015
Mixed	207	No effect	ER^−^; HER2^+^; TNBC	High grade, Ki67^+^	Papaioannou et al. 2019

Abbreviations: DCIS, ductal carcinoma in situ; ER, estrogen receptor; LN met^+^, lymph node involvement; ND, not determined; NS, no significant differences; PR, progesterone receptor; TIL, tumor-infiltrating lymphocytes; TNBC, triple-negative breast cancer.

a Not significant in multivariate analysis.

b Poor prognosis in low-CD8^+^ tumors.

c Favorable prognosis in high-CD8^+^ tumors.

d Compared to normal breast.
